# Inhibition effects of a natural inhibitor on RANKL downstream cellular signalling cascades cross‐talking

**DOI:** 10.1111/jcmm.13703

**Published:** 2018-06-17

**Authors:** Biao Wang, Dingjun Hao, Zhen Zhang, Wenjie Gao, Hu Pan, Yuan Xiao, Baorong He, Lingbo Kong

**Affiliations:** ^1^ Hong‐Hui Hospital Xi'an Jiaotong University College of Medicine Xi'an China

**Keywords:** BMMs, myricitrin, NFATc1, osteoclastogenesis

## Abstract

Myricitrin is a natural occurring flavonoid glycoside that possesses effects on inhibiting nitric oxide (NO) transmission and preventing inflammatory reaction. Although previous study showed the myricitrin possesses antibone loss effects via reducing the expression of IL‐6 and partially suppressing reactive oxygen species (ROS) production. However, the effects of myricitrin on nuclear factor‐kappaB ligand (RANKL)‐stimulated osteoclastogenesis have not yet been further investigated. The current study was aimed to demonstrating the inhibitory effects of myricitrin on RANKL‐stimulated osteoclastogenesis and relevant mechanisms. We found myricitrin significantly suppressed osteoclastogenesis suggesting that it may acts on RANKL/RANK induced downstream signal cross cascading in osteoclast precursors. In that, our Western blotting results showed myricitrin significantly attenuated RNAKL/MAPKs (phosphorylation of p38, ERK, JNK) and AKT signal cascading. Complementing previous study, our results suggesting as a natural inhibitor, myricitrin possesses the potential therapeutic effects on inflammatory osteolysis.

## 1 INTRODUCTION

Exploring the pharmaceutical natural agents, which could prevent the progression of bone loss disorders, we need to know the mechanisms involved in these natural agent's prevention effects underlying osteoclastogenesis (process of osteoclast differentiation). Osteoclastogenesis is primarily governed by 2 key cytokines, receptor activators of the nuclear factor‐κB (NF‐κB) ligand (RANKL) and macrophage colony‐stimulating factor (M‐CSF).^1^, ^2^ M‐CSF induces expression of RANKL receptor (RANK) as well as supports survival and proliferation of OCs lineages.^3^, ^4^ While RANKL interacts with the OCs surface receptor RANK, which, in turn, serves as a trigger of downstream signalling pathways for osteoclastogenesis.^5^ These signalling include three mitogen‐activated protein kinases (p38 MAPK, ERK and JNK) and AKT.^6^ Moreover, RANK activates the transcription–factor complex, activator protein 1 (AP1), through induction of its component c‐Fos, which in turn, auto amplified nuclear factor of activated T cells, cytoplasmic 1 (NFATc1), the master regulator of osteoclast differentiation.^7^, ^8^ Further, NFATc1 is a master factor that activates the expression of osteoclast marker genes and subsequently results in enhanced differentiation and function of osteoclasts.^9^ Taking together, interfering with these pathways may help prevent pathologically enhanced OCs formation and bone loss.

**Figure 1:**
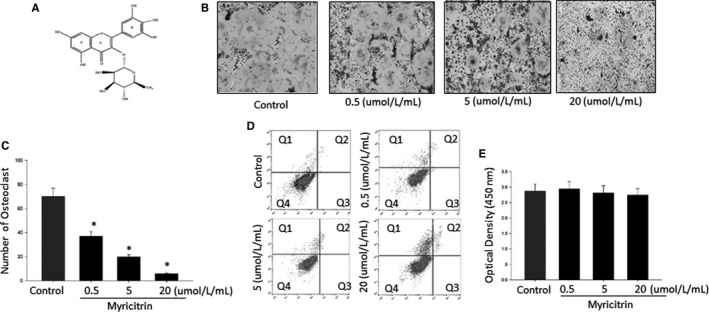
Chemical structure of Myricitrin and Effect of Myricitrin on RANKL‐induced OCs differentiation. A, Chemical structure of Myricitrin; B, TRAP‐positive cells were counted as osteoclasts in each experimental group. C, Relative number and area of TRAP‐positive osteoclasts. Asterisk indicates a statistically significant difference (*P <* .05) between control and treated. D, Bone marrow macrophage cells treated with myricitrin concentrations for 48 h were stained with Annexin V‐PE and 7‐AAD and FACS used to determine the percentage of dead and apoptotic cells (Q2 and Q3) within each population. E, Cell viability was determined by XTT assay. Asterisk indicates a statistically significant difference (*P <* .05) between control and treated. Similar results were obtained in at least 3 independent experiments

Natural product myricitrin (Figure 1) is a botanical flavone and has been widely used as a folk medicine in China.^10^ Several studies reported myricitrin possesses effective antioxidative,^11^ anti‐inflammatory^12^ and can protect a variety of cells from in vitro and in vivo injuries.^13^, ^14^ Otherwise, recently studies have showed myricitrin regulates various cellular signalling cascades including STAT3^15^ and PI3K/Akt/eNOS.^16^ Moreover, myricitrin demonstrated suppression effects on myocardial apoptosis relied on the ERK/p53‐mediated mitochondrial apoptosis pathway.^17^ Furthermore, the most recently study showed the protective effects of myricitrin against osteoporosis via reducing the expression of IL‐6 and partially suppressing ROS production.^18^ Nevertheless, as dairy natural occurring inhibitor, deeply exploring the novel effects of myricitrin on osteoclastogenesis still on the way. In our current study, we aimed to demonstrate the cellular effects of myricitrin on osteoclastogenesis and precise mechanisms underlying.

## 2 MATERIALS AND METHODS

### 2.1 Reagents and antibodies

Myricitrin was obtained from ChemFaces(Wuhan, China, CAS No.17912‐87‐7). Recombinant human macrophage colony‐stimulating factor (M‐CSF) and human RANKL were obtained from PeproTech EC, Ltd. (London, UK). Rabbit antibody against NFATc1 was purchased from Cell Signaling Technology, Inc. (Danvers, MA, USA). Rabbit antibody against c‑Fos was purchased from Santa Cruz Biotechnology, Inc. (Dallas, TX, USA). The XTT assay kit was obtained from Roche (Indianapolis, IN, USA). Western blot antibodies for phosphor‐AKT, phosphor‐ERK, ERK, phosphor‐JNK, JNK, phosphor‐p38 and p38 were from Santa Cruz Biotechnology Inc. (Santa Cruz, CA, USA); β‐actin antibody was purchased from Sigma‐Aldrich, Inc. (St. Louis, MO, USA).

### 2.2 OCs differentiation

All animal care and experimental protocols were approved by the “Medical Ethics Committee of Hong‐Hui Hospital, Xi'an Jiaotong University School of Medicine” (No.1002016011) and performed strictly according to the “Guidelines of Jiaotong university institutes of Health for the care and use of laboratory animals.” Bone marrow macrophage cells (BMMs) culture, osteoclast differentiation methods and myricitrin dose treating were following previous studies.^16^, ^19^, ^20^ Briefly, by flushing the femurs and tibiae of 5‐week‐old ICR mice with α‐minimum essential medium and suspended in α‐MEM supplemented with 10% foetal bovine serum. Nonadherent cells were collected and cultured for 3 days in the presence of M‐CSF (20 ng/mL). Floating cells were discarded, and adherent cells on dish bottoms were classified as BMMs. BMMs were seeded at 3.5 × 10^4^ cells/well in α‐MEM/10% FBS and were cultured in the presence of M‐CSF (20 ng/mL) and RANKL (40 ng/mL) for 4 days in the presence or absence of myricitrin (0, 0.5, 5 or 20 μmol/L/mL). TRAP‐positive multinucleated cells with greater than three nuclei were counted as OCs.

### 2.3 Cell viability assays and cell apoptosis assay

Cell viability and apoptosis of BMMs after myricitrin (0, 0.5, 5 or 20 μmol/L/mL) treatments was studied by XTT (Indianapolis, IN, USA) and flow cytometry as previous studies reported, respectively.^21^ Briefly, BMMs were seeded in 96‐well plates by the 1 × 10^4^ cells with different concentrations of myricitrin. XTT solution was added to each well and incubated for 4 hours. The plate was read at 450 nm. Meanwhile, after the myricitrin‐treated BMMs for 48 hours, the cells then stained by Annexin V‐PE and 7 AAD for 15 minutes. Subsequently, cell apoptosis were assessed by excited at 488 nm and signals from 10 000 cells acquired at 585/42 (564‐606 nm) and 702/64 (670‐735 nm) in a FACS Canto II (BD). Results were analysed by the FACSDiva (BD) software and expressed as the percentage of apoptosis cells within each population.

### 2.4 Bone absorption assay and confocal‐microscopy immunofluorescence

Bone absorption assay and filamentous actin immunofluorescence staining method were following previously described.^22^ Briefly, BMMs were seeded onto bovine bone slices with three replicates. After culturing for 48 hours at 37°C, cells were stimulated with 40 ng/mL RANKL and 20 ng/mL M‐CSF with or without myricitrin treatment (0, 0.5, 5 or 20 μmol/L/mL) until mature osteoclasts formed. Cells were removed by mechanical agitation and sonication. Resorption pits were visualized by Philips XL30, and the percentage of bone resorption area was quantified using Image J software (NIH, Bethesda, MD, USA). In addition, for the filamentous actin, myricitrin‐treated BMMs cultured for 4 minutes at room temperature then fixed in 4% paraformaldehyde after washing in phosphate‐buffered saline (PBS). Then, cells were incubated with 2 units/mL phalloidin and DAPI solution for visualization of filamentous actin, 25°C for 1 hour. The fluorescence signal was observed using a laser scanning confocal microscope (Olympus FV1200; Olympus, Shinjuku, Japan), and images representative of 5 experiments were analysed using Image‐Pro Plus software (Media Cybernetics Inc. Rockville, MD, USA).

### 2.5 RT‐PCR

Total RNA was isolated with QIAzol reagent (QIAGEN, Valencia, CA, USA) according to the manufacturer's instructions RNA (1 μg) was reverse transcribed using oligo dT primers (10 μg) and dNTPs (10 mmol/L). The mixture was incubated at 65°C for 5 minutes, and cDNA was produced by incubating at 42°C for 50 minutes with first strand buffer (50 mmol/L Tris–HCl, pH 8.3, 75 mmol/L KCl, 3 mmol/L MgCl_2_), 100 mmol/L DTT, RNase inhibitor, and Superscript II reverse transcriptase (Invitrogen).The mouse GAPDH gene was used as internal control. The amplification parameters consisted of an initial denaturation step at 95°C for 5 minutes followed by 40 cycles of denaturation at 95°C for 1 minute, annealing at 60°C for 30 seconds and extension at 72°C for 1 minute. The specificity of the SYBR green assays was confirmed by melting‐point analysis. Expression data were calculated from the cycle threshold (Ct) value using the Ct. Primers employed for amplification are shown in Table 1.

**Table 1:** Primer sequences used for real‐time RT‐PCR analysis

Gene name	Primer sequence (5′‐3′) forward	Primer sequence (5′‐3′) reverse
c‐Fos	5′‐CTGGTGCAGCCCACTCTGGTC‐3′	5′‐CTTTCAGCAGATTGGCAATCTC‐3′
NFATc1	5′‐CTCGAAAGACAGCACTGGAGCAT‐3′	5′‐CGGCTGCCTTCCGTCTCATAG‐3′
GAPDH	5′‐TCA AGA AGG TGG TGA AGC AG‐3′	5′‐AGT GGG AGT TGC TGT TGA AGT‐3′

### 2.6 Western blotting

Protein lysates from myricitrin treated cells were prepared in a buffer containing 50 mmol/L with 50 μg/mL phenylmethylsulfonyl fluoride (Thermo Scientific, Waltham, MA, USA). Thirty micrograms of total cell proteins was mixed with loading buffer and separated on 10% SDS‐PAGE gels, and the proteins in the gels were electro‐transferred onto nitrocellulose membranes (GE, Marlborough, MA, USA). Horseradish peroxidase (HRP) conjugated secondary antibodies were used to visualize bands under an ECL‐based imaging system. The membranes were blocked with 5% nonfat milk in Tris‐buffered saline contacting 0.1% Tween‐20 (TBST) for 1 hour, before blotting with the primary antibodies for 2 hours at room temperature. The membranes were washed in TBST and incubated for 1 hour with HRP‐conjugated immunoglobulin antibodies. Signals were analysed in ImageJ and compared to controls after normalization.

### 2.7 Statistical analysis

Experiments were conducted separately at least 3 times, and all data are presented as the mean ± standard deviation (SD). All data analysis was performed using SPSS software package ver. 14.0 (SPSS, Chicago, IL); one‐way ANOVA was used for comparison among the different groups. Post hoc testing of differences between groups was performed by using Duncan's test when the ANOVA was significant. All results were considered to be significant at the 5% critical level (*P <* .05).

## 3 RESULTS

### 3.1 Myricitrin inhibits TRAP‐positive cells differentiation

Our cellular culture results showed, following M‐CSF plus RANKL treatment, myricitrin significantly decreased the TRAP‐positive multi nucleated OCs formation in the dose‐dependent manner (Figure 1B). Otherwise, we performed XTT assay and flow cytometry to investigate the cytotoxicity and cellular apoptosis, respectively. Both methods showed, after incubation with myricitrin at the same doses which effectively down‐regulated OCs formation, our results demonstrated no cell death was significantly increased by myricitrin treatment (Figure 1E,D), demonstrating that the inhibitory effects of myricitrin on osteoclastogenesis were not related to cellular toxicity.

### 3.2 Myricitrin decreases mature OCs bone resorption in vitro

**Figure 2:**
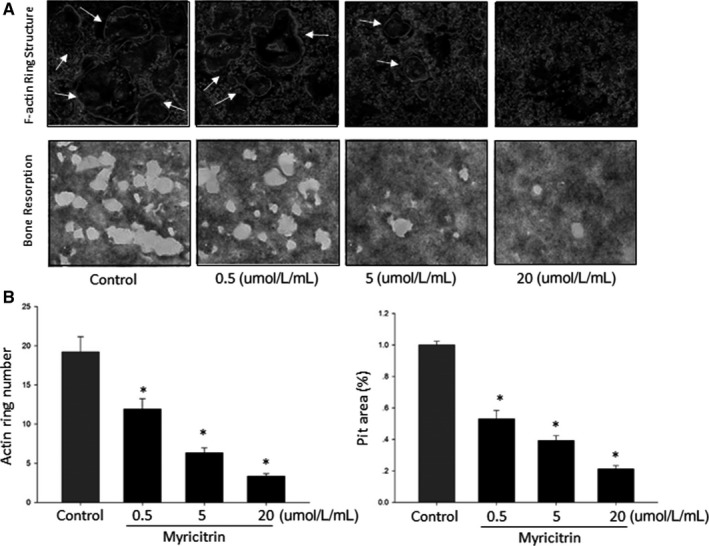
Myricitrin inhibited osteoclast bone resorption and F‐actin ring structure. A, Representative scanning electron microscopy images of F‐actin ring structure and bone resorption pits; B, Number of F‐actin ring and bone resorption pits. All experiments were performed at least three times, and the significance was determined as indicated in methods (*P < .05)

To investigate the effects of myricitrin on osteoclastic bone resorption, BMMs were cultured onto bovine bone slices, after attachment, cells culturing without or with various concentrations of myricitrin (0.5, 5 and 20 μmol/L/mL). As results shown, after myricitrin treatment, the pit's number and size of bone resorption were significantly down‐regulated. (*P <* .05). Complemented to above bone resorption pit study, otherwise, the ring‐liked sealing zone of filamentous actin ring formation is a key indicator of osteoclast formation. Consist with resorption pits electron microscopy scanning results, after treatment with myricitrin, both number and morphology of the ring‐liked sealing zone of filamentous actin were down‐regulated by myricitrin treatment (Figure 2). Considering above two studies reflect the functional regulation effects of myricitrin on mature OC, we hypothesized that myricitrin could exert therapeutic effects on bone loss by intervene OC's resorptive activity.

### 3.3 Myricitrin attenuating c‐Fos/NFATc1 expression

**Figure 3:**
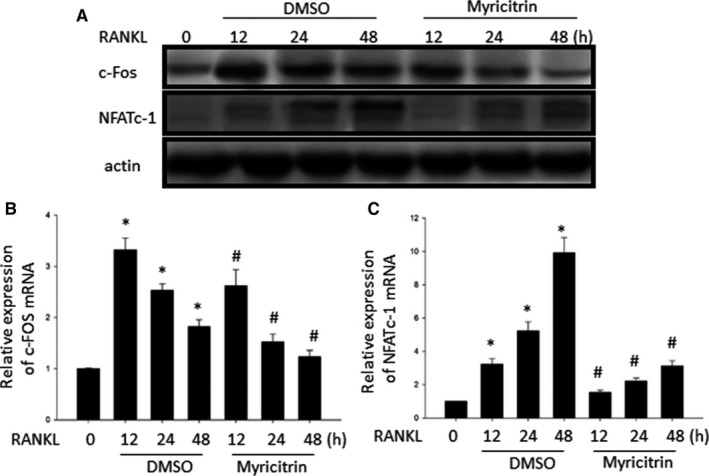
Effect of Myricitrin on RANKL‐induced the mRNA expression of c‐Fos and NFATc1. A, Western blotting analysis showed myricitrin inhibits the expression of c‐Fos and NFATc1 induced by RANKL. The intensities of the protein bands were analysed and normalized to actin. Similar results were obtained in at least 3 independent experiments. B and C Bone marrow macrophage cells were pretreated with or without myricitrin (5 μmol/L) for 1 h and with RANKL (100 ng/mL) for the indicated time (*P < .05 vs. Sham; ^#^ *P* < .05 vs. DMSO). The mRNA expression of c‐Fos and NFATc1 genes was analysed by real‐time RT‐PCR

As aforementioned, c‐Fos/NFATc1 as two critical transcription factors participate the late stage of osteoclastogenesis.^7^ Therefore, to explore the underlying mechanisms of down‐regulation effects of myricitrin on osteoclastogenesis, we initially focus on study of the effects of myricitrin on c‐Fos/NFATc1. Our results demonstrated that, RANKL significantly induced c‐fos and NFATc1 activity by manifested in increased in mRNA and protein level. However, as we expected c‐Fos/NFATc1 expression was significantly decreased by myricitrin treatment (Figure 3). These results manifested c‐fos/NFATc1 at least as one target involved in the inhibitory effects of myricitrin on osteoclastogenesis.

### 3.4 Myricitrin inhibits osteoclastogenesis via the suppression of MAPKs and AKT phosphorylation

**Figure 4:**
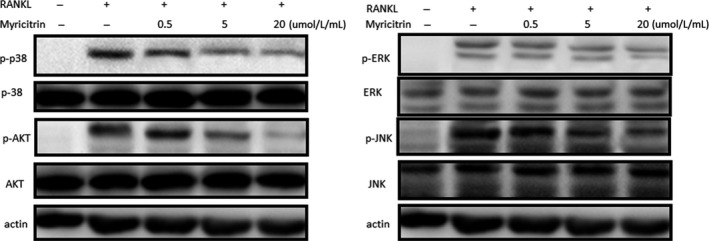
Myricitrin inhibits osteoclastogenesis via the suppression of MAPKs and AKT phosphorylation. Myricitrin down‐regulated the MAPKs and AKT phosphorylation in RANKL‐induced bone marrow macrophage cells. The intensities of protein bands were analysed and normalized to actin. Similar results were obtained in at least 3 independent experiments

However, during the osteoclastogenesis process, c‐fos/NFATc1 axis co‐stimulated by various signaling cascades, such as MAPKs and AKT signalling.^23^ Thus, to validate the upstreaming signalling pathway that cross‐talking in the inhibitory effects of myricitrin on transcription factors (c‐Fos/NFATc1) expression, we explored the effects of myricitrin on RANKL/MAPKs and RANKL/AKT signalling pathways. Our results showed that phospho‐ERK, phospho‐JNK, phospho‐p38 MAPK and phospho‐AKT were suppressed by myricitrin in a concentration‐dependent manner (Figure 4).

## 4 DISCUSSION

Osteoporosis commonly caused by enhanced pathological bone resorption or bone loss because of the increased OCs activation.^24^ Previous study proved excessive OCs differentiation (osteoclastogenesis) is mostly via the classic signalling pathway: RANKL, which has been a potential valuable target for treating osteoporosis.^5^ Our current study of myricitrin down‐regulation effects on osteoclastogenesis and decreased mature OC's bone resorptive function have demonstrated myricitrin as a natural inhibitor, possessing therapeutic potential on treating pathological bone loss disease. Moreover, complementary to previous study, we further investigated the effects of myricitrin on c‐fos/NFATc1 and its upstreaming signalling cascades such as MAPKs and AKT, during osteoclastogenesis.

During the past decade, most large pharmaceutical companies ceased natural products (compounds/agents/inhibitors) screening, which is initial step for drug discovery.^25^, ^26^ Despite the lack of effort by most of the large pharmaceutical companies, natural product research has still been active during the past decade.^27^, ^28^ Institutions mainly from China, Korea, India, Japan and United States keep engaging in the work of natural products study, for re‐establish natural products as a major source for potential drug discovery.^19^, ^26^, ^29^, ^30^, ^31^ Given that only limited biological properties or functional activities have been worked out for each natural product, there still has a “long way to go” for fully identifying the biological activities of natural products.^33^, ^34^ Myricitrin is a naturally occurring flavonoid derived from bayberry bark and fruit, which has been reported to exhibit numerous biological activities such as antioxidative, anti‐inflammatory and antinociceptive effects.^12^, ^14^, ^35^ Despite the biological activities, myricitrin is relatively easy to extract and purify.^15^

Documents studies showed myricitrin regulates several extracellular signalling pathways, such as STAT3, PI3K/Akt/eNOS^15^ and ERK/p53^17^ The most recently study showed myricitrin inhibited osteoporosis of ovariectomized rats, which was associated with reducing the expression of IL‐6 and partially suppressing ROS production.^18^ In complementary to these findings, we performed F‐actin ring formation and bone absorption assay in vitro to explore the inhibitory effects of myricitrin on osteoclast differentiation and further investigate the underlying mechanisms involved in these down‐regulation effects. Our results showed both impaired F‐actin ring formation and enlarged bone resorption pits were dramatically decreased by myricitrin which consistently with our previous in vitro study.

RANKL interacts to its receptor RANK is a pivotal upstreaming signal pathway involved in osteoclastogenesis, further triggers various downstreaming signalling cascades, such as MAPKs (p‐38, ERK and JNK) and Akt, which leading to stimulate the activation of critical genes for osteoclastogenesis.^7^, ^36^ In addition, paralleling RANKL/RANK signalling, ROS directly affect osteoclastogenesis by increasing TNF‐α and IL‐6, which in turn indirectly up regulates RANKL expression.^37^ Previously, Huang et al^18^ reported that myricitrin inhibited RANKL expression in MC3T3‐E1 cells under oxidative conditions, we hypothesized this merit might be attributed to the polyphenol structure: 3′4′5′‐trihydroxyl‐substituted B ring of myricitrin. Otherwise, complementary to above study, our current results for the first time demonstrate myricitrin inhibit osteoclastogenesis by directly attenuated the c‐Fos/NFATc1 expression followed by inhibiting RANKL/MAPKs and RANKL/AKT signalling cascades.

In that, as previous study reported, phosphorylation‐ERK is the crucial regulator of AP‐1 (c‐Fos and c‐JUN) activation in BMMs.^38^ Phosphorylation of c‐Fos is sustained by ERK signalling, impaired ERK activation will indirectly leading the attenuation of c‐Fos.^39^ p38 is particularly critical for the OCs early stage differentiation, as it promotes the activity of microphthalmia‐associated transcription factor (MITF) and TRAP expression.^6^ AKT signalling is crucial in OCs survival.^40^ Previous studies showed the importance of the AKT/NFATc1 signalling cascades in OCs differentiation.^41^ Therefore, combined with the results of our Western blot results, we speculate MAPKs and AKT cascades might be an underlying mechanisms involved in the regulation of myricitrin on osteoclastogenesis. However, PI3K and STAT3 are two up signal transduction activators of AKT during advanced cell survival stage.^42^ Thus, further investigations of myricitrin on PI3K and STAT3 effects during OC differentiation should necessarily be conducted.

In sum, our current study demonstrated the inhibitory effects of myricitrin on osteoclastogenesis in vitro and therapeutic effects in pathological osteoporosis in vivo, respectively. Moreover, our study explored myricitrin consequently attenuates the activation of the OCs‐specific transcription factors c‐Fos/NFATc1. Further, we identified that myricitrin functioned by attenuating the RANKL/MAPKs signalling pathway cross‐talking RANKL/AKT signalling cascades. Combined with previous studies, our current results indicate myricitrin are promising candidates for the treatment of pathological bone loss diseases.

## CONFLICT OF INTEREST

The authors declare that they have no competing interests.

## CONSENT FOR PUBLICATION

The manuscript is approved by all authors for publication.

## AVAILABILITY OF DATA AND MATERIALS

All data and materials were included in the manuscript.
